# SINGING WITH DEMENTIA: CREATING AND PILOT TESTING A VIDEO TO CHANGE ATTITUDES OF MEDICAL RESIDENTS

**DOI:** 10.1093/geroni/igad104.2657

**Published:** 2023-12-21

**Authors:** Jody Sharninghausen, Carrie Ann Davison, Richard Marottoli

**Affiliations:** Yale School of Medicine, New Haven, Connecticut, United States; Yale School of Medicine, New Haven, Connecticut, United States; Yale Medical School, New Haven, Connecticut, United States

## Abstract

As part of the “Creativity in Aging” project at Yale Geriatrics, we are making a series of short videos highlighting individuals and programs that transcend dementia through music. The target audience includes health trainees and professionals who care for older adults. The first video, made by a PhD candidate and an internal medicine resident physician, features The Recollections: a chorus of community dwelling people with dementia, their caregivers, and college students. Video learning objectives are to 1) change dementia attitudes; 2) demonstrate preserved skills and abilities of persons with dementia; and 3) raise awareness about local initiatives for music in dementia and how to refer patients. We interviewed the chorus founder, a geriatric psychiatrist, who described the creative process, including logistics and vision, and provided advice to caregivers and medical residents. Live clips from chorus rehearsals were shown, including singing and dancing, with voiceovers describing participant stories. Slides reinforced key points. The video was pilot tested on a group of residents from the Internal Medicine and Psychiatry programs. All respondents (N=6) used positive words such as “optimistic” and “inspired” to describe how the video made them feel. Most indicated it was moderately relevant to their care of patients with dementia and several provided suggestions on how to increase relevance. Constructive feedback included parts to compress or expand, overall length, visual aspects, and desire for more ‘how to’ guidance. Future plans include sharing a revised version with the broader residency program with pre-and-post dementia attitudes surveys.

